# Cellular Environment and Phenotypic Heterogeneity: How Data-Driven Modeling Finds the Smoking Gun

**DOI:** 10.3390/ijms23126536

**Published:** 2022-06-10

**Authors:** Marie Guilbert, Emmanuel Courtade, Quentin Thommen

**Affiliations:** 1 CNRS, UMR 8523-PhLAM-Physique des Lasers Atomes et Molécules, University of Lille, F-59000 Lille, France; marie.guilbert@univ-lille.fr (M.G.); emmanuel.courtade@univ-lille.fr (E.C.); 2 CNRS, Inserm, CHU Lille, Institut Pasteur de Lille, UMR9020-U1277-CANTHER-Cancer Heterogeneity Plasticity and Resistance to Therapies, University of Lille, F-59000 Lille, France

**Keywords:** extracellular matrix, phenotypic heterogeneity, cellular stress response, heat stress, single cell, mathematical modeling, time lapse microscopy

## Abstract

The cellular environment modifies cellular phenotypes, in particular, the stress response phenotype, which easily exhibits high phenotypic heterogeneity due to the common characteristics of its regulatory networks. The aim of this work is to quantify and interpret the impact of collagen type I, a major component of the cellular environment, on the phenotypic heterogeneity of the cellular response. Our approach combines in an original way the monitoring of the response of a single cell and the mathematical modeling of the network. After a detailed statistical description of the phenotypic heterogeneity of the cellular response, the mathematical modeling explains how the observed changes can be explained by an induced increase in the average expression of a central protein of the regulatory network. The predictions of the data-driven model are fully consistent with the biochemical measurements performed. The framework presented here is also a new general methodology to study phenotypic heterogeneity, although we focus here on the response to proteotoxic stress in HeLa cells.

## 1. Introduction

Cellular phenotypes are highly dependent on surface receptor interactions with the ExtraCellular Matrix (ECM) [1] that modulate cell adhesion, migration, proliferation, survival, and, more generally, signaling. Type I collagen, which represents the major component of the ECM in the body’s connective tissues, is the most widely used protein in cell culture engineering to study living cells under more physiological conditions and to mimic their in vitro microenvironment [2]. Even with a monoclonal cell line and a homogeneous environment, cellular phenotypes vary from one cell to another, noting responses to external perturbation [3]. The origin of this phenotypic heterogeneity has been the subject of much debate, but one of the causes frequently mentioned concerns the stochasticity of molecular processes, their intrinsic noises, which would contribute to generate molecular differences between cells. Phenotypic heterogeneity is often presented as a possible origin of cell fate divergence within a population, and therefore, as an important phenomenon to characterize in order to best control this issue [4]. Phenotypic heterogeneity is thus regularly pointed out in the context of antitumor therapies, whether it is fractional killing [5], resistance [6], or any other phenomenon leading to tumor resurgence [7]. Until now, this phenotypic heterogeneity has mainly been studied in the absence of an extracellular environment. Given its potential importance on cell fate in physiological conditions, whether phenotypic heterogeneity is not over or underestimated due to the absence of ECM deserves more detailed investigation. Since cell fate is regulated by signaling pathways, and thus, among others by cellular responses to stress, we chose to study phenotypic heterogeneity in this context. A particularity of cellular responses to stress is the activation of stress-inducible scavengers in a hypersensitive rather than gradual manner, this particularity allows us here to finely measure the variation induced by the culture condition, as it amplifies the phenotypic heterogeneity. Among the multiple cellular responses to stress is the Heat Shock Response (HSR), or HSF1-mediated proteotoxic stress response, which forms a protein quality control system that repairs, refolds, or targets damaged proteins for degradation [8]. In this context, it has recently been shown how hypersensitive activation combined with variability in scavenger expression induces a continuum of responses, making the cellular response to heat stress a system of choice for studying phenotypic heterogeneity [9].

The aim of this study is therefore to investigate the influence of the extracellular matrix on the phenotypic heterogeneity of the cellular response to stress by focusing on the cellular response to heat stress and the collagen type I coating. We combine high-throughput measurements and novel mathematical modeling methods to quantify and analyze phenotypic variability under different culture conditions. In particular, the heat stress response (HSR) was chosen for four reasons: (1) HSR can be easily stimulated in a homogeneous and temporally controlled manner by sample temperature; (2) HSR dynamics can be quantitatively monitored by time-lapse microscopy [9,10]; (3) a mathematical model based on HSR data has been developed [11]; and (4) the molecular origin of the phenotypic heterogeneity of HSPs has been demonstrated [9]. First, we investigate the impact of collagen type I coating on the variability of protein expression of three essential HSR players: the transcription factor HSF1 and two major members of the 70-kDa heat shock protein (HSP70) family, which are known to control HSR phenotypic heterogeneity. Then, in a second step, high-throughput time-lapse microscopy is used to track and statistically analyze the impact of collagen type I coating on the phenotypic heterogeneity of HSR. Finally, mathematical modeling based on data extracted from single-cell dynamics is used to predict the variability of protein expression in agreement with the experimental measurements obtained in the first step. Overall, these results reveal that collagen type I coating only slightly modifies phenotypic variability. They also interestingly reveal that tuning occurs through protein expression which can be reconstructed by their dynamics through a quantitative mathematical model.

## 2. Results

### 2.1. More Than Cell Cycle Phase, ECM Affects the Protein Expression Level and Variability

**Figure 1:**
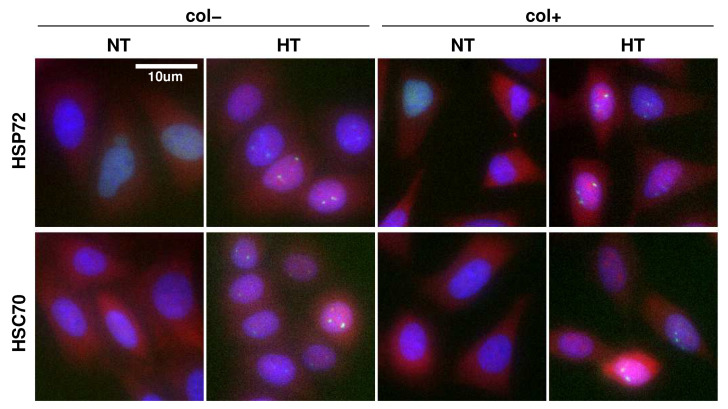
Immunofluorescence staining of HSPs and HSF1. HeLa-HSF1:eGFP cells exposed to normothermia (NT) at 37 °C or hyperthermia (HT) consisting of a one hour at 43 °C for two different culture conditions: with collagen coating (col+) or not (col−). HSP (red channel), HSF1:eGFP (green channel), Hoechst (blue channel). In HT, bright green spots localized in the nucleus correspond to HSF1 foci.

**Figure 2:**
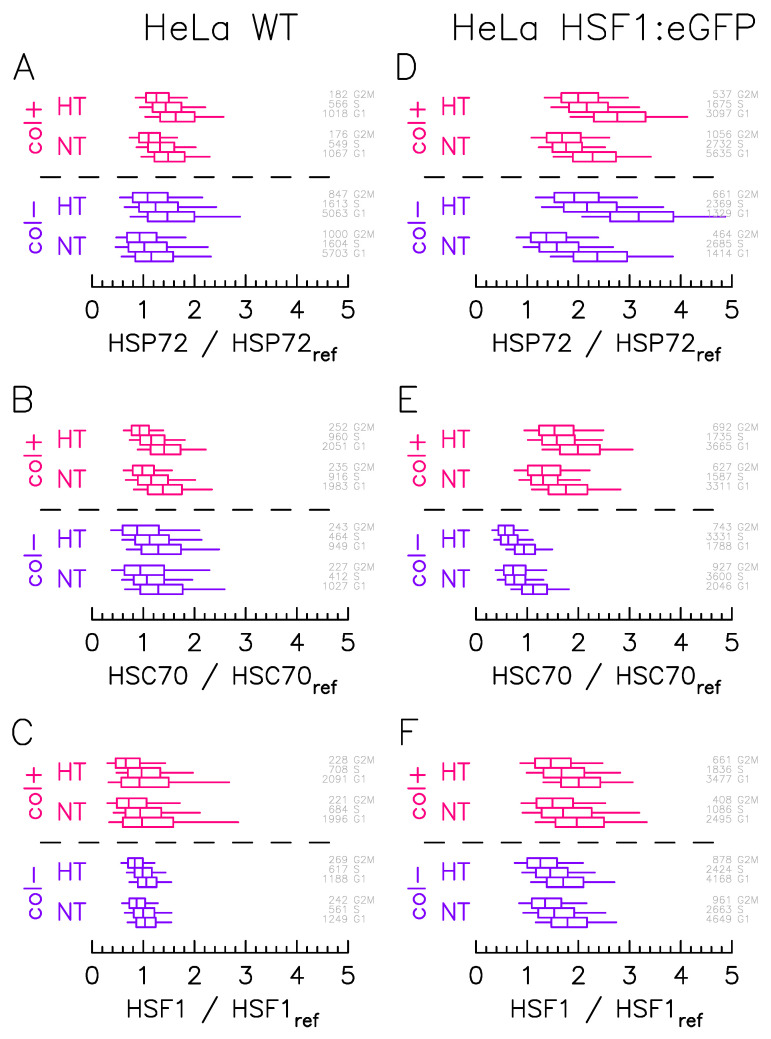
Culture condition affects the protein expression level distributions more than cell cycle phase. HSP2, HSC70, and HSF1 protein expression distributions measured by immunofluorescence in (**A**–**C**) HeLa Wild Type and (**D**–**F**) HeLa–HSF1:eGFP cell lines for in case of NormoThermia (NT: 37 °C) or HyperThermia (HT: 1 h at 43 °C). The boxes represent quartile and the whiskers represent the 9th percentile and the 91st percentile. The color indicates the culture condition: blue for cells adhered to non-coated (col−); red for cells adhered to collagen-coated (col+). Cell cycle analysis on Hoechst fluorescence signals is used to cluster cells by cell-cycle stage (G1, S, G2/M), cell number and cell-cycle stage are indicated in gray directly on the right left of each the box plot. The fluorescence level is normalized to the Hoechst fluorescence signal and the reference level ($\mathrm{HSF}1_{\mathrm{ref}}$, $\mathrm{HSP}72_{\mathrm{ref}}$, or $\mathrm{HSC}70_{\mathrm{ref}}$) corresponds to the median renormalized fluorescent value measured on HeLa WT cell line in G1 phase at 37 °C for cells adhered to non-coated.

We first precisely quantify the protein expression variability from immunofluorescence experiments (Figure 1). The quantification study is carried out on samples exposed to NormoThermia (NT) at 37 °C or HyperThermia (HT) consisting of a one hour at 43 °C. Two different culture conditions are performed: presence of ECM made of collagen coating (col+) or not (col−). The quantification procedure concerns, in addition to the transcription factor HSF1, the two main molecular chaperones of the HSP70 family: HSP72 (HSPA1A), a stress inducible chaperone and HSC70 (HSPA8), a constitutively expressed one (Figure 1) [12]. In order to normalized each fluorescence measurement to a reference value, we use here the median expression in culture condition of Hela WT cells. The protein expression variability displays log-normal distributions that are here represented by box and whisker plots, the number of counting cells being in gray at the right (Figure 2). Interestingly, the expression variability can additionally be segmented according to the cell cycle phases (S, G1 and G2+M) via Hoechst labeling in immunofluorescence protocol. Cell cycle phases have indeed also been proposed as source a major of protein expression variability. If the HF1:eGFP insertion eases activation follow-up in living cells through time lapse microscopy, it also induces a genetic modification potential extra-source of expression variability. So, we quantified the distributions in the genetically modified HeLa cell line (Figure 2D–F), but also in the wild type one (Figure 2A–C).

As a first result, the segmentation according to the cell cycle phase does not reveal any notable difference. In each case, distributions mostly overlap in different phases. As expected, the G1 phase distribution shows a higher median level of expression. This increase appears more pronounced in the HeLa HSF1:eGFP line for HSP72 (Figure 2D). Still expected is the HSP72 overall expression increase upon HT as this molecular chaperone is known to be stress inducible (Figure 2A–D) whereas HSC70 (Figure 2B–E) and HSF1 (Figure 2C–F) overall expressions does not vary. The stretching of HSF1 (Figure 2F), and consequently of HSP72 (Figure 2D), expression heterogeneity in the presence of the HSF1:eGFP insertion has been previously observed [9] and is confirmed here in the presence of the ECM.

More surprisingly, and also more interestingly for the issue at hand, is the modification of expression levels depending on the culture condition. In HeLa WT cells, the presence of the ECM indeed tends to stretch, in basal condition (NT), the expression level of HSF1 (Figure 2C), and to increase the HSP72 expression level (Figure 2A). The same effect is observed with HeLa HSF1:eGFP modified cells, to a lesser extent for HSP72 (Figure 2D) and HSF1 (Figure 2F) as may be masked by the stretching of heterogeneity due to insertion. Surprisingly, HSC70 expression level is significantly enhanced in the presence of ECM (Figure 2E).

### 2.2. ECM Does Not Affect the Foci to Protein Expression Activation Function

Let us now investigate how protein expression variability affects the stress response heterogeneity. It is known that the foci intensity, meaning the fraction of HSF1 fluorescence located within foci and here named *F*, is a valid quantitative reporter of the HSR activation [9]. However, it has also been shown that *F* sharply decreases with protein expression level and specifically on molecular chaperones to transcription factor HSF1 expression ratio (HSP72/HSF1 or HSC70/HSF1). The more the molecular chaperon expression or the less the HSF1 expression, the less is the fluorescence within foci. In our experiment, the *F* value displays a huge cell-to-cell variability that reflects activation heterogeneity whereas the stress condition is similar for all cells. The presence of the ECM may impact the response heterogeneity via the expression level tuning, as seen in the previous section, but also via a modification of the response activation dependency on expression level (either shape change or threshold shift).

**Figure 3:**
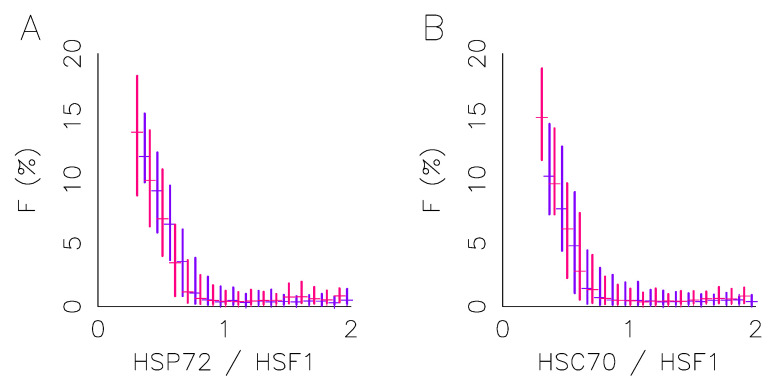
Foci intensity varies with protein concentration. This figure focuses on foci intensity after one hour at a 43 °C temperature in HeLa-HSF1:eGFP cell lines obtained by immunofluorescence targeting HSP72 and HSC70, respectively. Foci intensity distribution (box plot) vs. HSP72 to HSF1 ratio (**A**), or HSC70 to HSF1 ratio (**B**). (Blue) cells adhered to non-coated, (red) cells adhered to collagen-coated.

However, we checked here that the response activation function is unaffected by the presence or absence of the ECM (Figure 3). The fraction of HSF1 fluorescence within foci (*F*) is quantified in each cell on immunofluorescence images obtained with the HeLa-HSF1:eGFP cell line after one hour of exposure at 43 °C (see Figure 1 for an example of foci upon stress). Therefore, we may correlate, for each cell, the *F* measurements, with the expression level of the transcription factor HSF1, and the expression level of the molecular chaperone. It has been previously shown how *F* depends on the expression ratio. We thus segment the data according to the value of the expression ratio and calculate quartiles of the *F* distribution in each ratio class in order to represent the whole data set in a concise way. Regardless of the molecular chaperone considered, the activation function appears to be identical in both culture conditions. The vertical lines in Figure 3 represent the interquartile range, in red for the culture on collagen and in blue without, the median value is also indicated. Note that only classes with more than 30 cells are shown.

Figure 3 also confirms the extent of the heterogeneity of the response where spatial homogeneity of the temperature field was well controlled and not spatial. Indeed, the stress condition is identical for each cell, but a cell may present, or not, fluorescence foci; and the fraction of fluorescence localized in these foci varies continuously up to 20%. By setting a threshold for cell discrimination at 1% of fluorescence localized in foci, we obtain a rate of cells responding around 50%. Neither this response rate nor the shape of the response function depends on the cell cycle phase determined by the Hoechst fluorescence. All this suggests that the continuum of response activation is driven by the protein expression heterogeneity in a similar manner, whatever the culture condition, even if expression heterogeneity may differ with it. To further study the impact of the ECM on the cell response and its heterogeneity, we then conducted time-lapse microscopy experiments to follow the response dynamics over the duration of the stress application at the single cell level.

### 2.3. Monitoring Response Activation on HeLa Single Cell Level

HeLa cells were exposed to proteotoxic stress performed by an applied transient hyperthermia (three hours of exposure). The exposure temperature of the HyperThermia sequence (HT) modulates the intensity of proteotoxic stress, at 41 °C, 42 °C, 43 °C, and 44 °C. To unveil the ECM impact on response variability, the same protocol was applied on two cell samples corresponding to two culture conditions, one with collagen coating (col+) and the other without (col−). Finally, the cellular response to stress was timely tacked in each cell of the layer by fluorescence time lapse microscopy. The HeLa cells used were transfected with HSF1:eGFP, and the activation of the stress response is reflected by the appearance of fluorescence foci within the cell nuclei corresponding to the Nuclear Stress Bodies.

**Figure 4:**
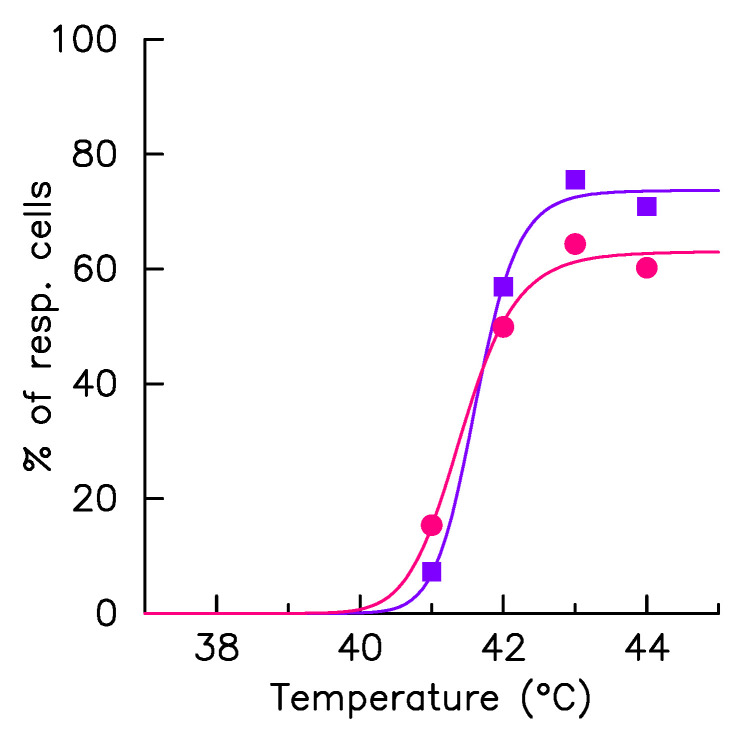
Fraction of responding cells. The HSF1:eGFP fluorescence and fraction of HSF1:eGFP fluorescence in nSBs (*F*) are monitored over time in a single cell upon a 41–44 °C heat stress, time zero coincides with the stress onset. The fraction of cells having detectable fluorescence foci during the hyperthermia phase (dots) are displayed as function of the exposure temperature in col+ (red) and in col− (blue) conditions. The solid lines, which correspond to the result of the adjustment by a Hill function, serve only as eye guidance.

**Figure 5:**
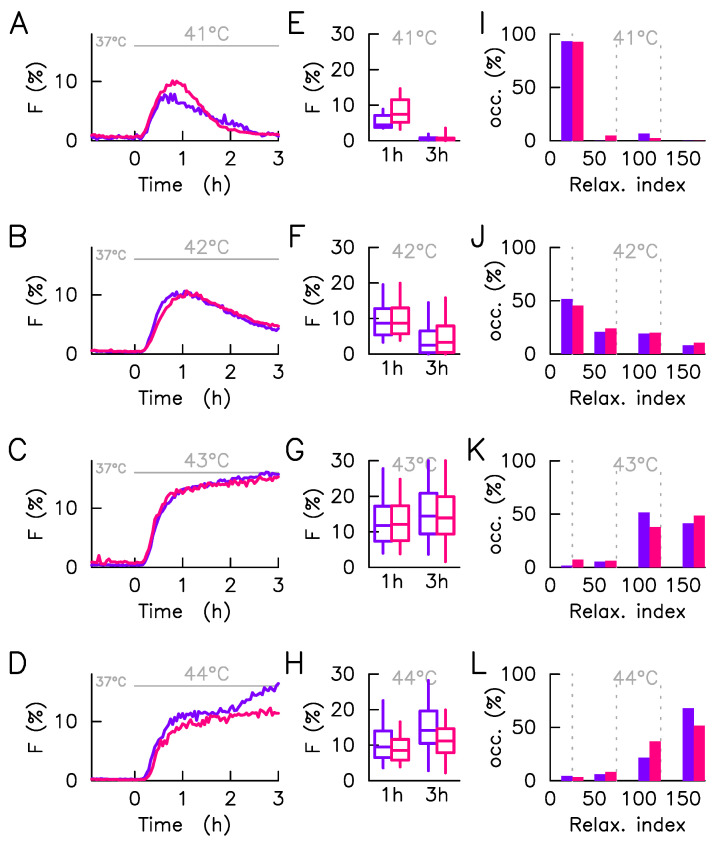
Cell-to-cell variability of responding cells in heat shock response. The HSF1:eGFP fluorescence and fraction of HSF1:eGFP fluorescence in nSBs (*F*) are monitored over time in a single cell upon a 41–44 °C heat stress, time zero coincides with the stress onset. Each line of the plot corresponds to a given stress condition. In all panels, measurements obtained with cells adhered to non-coated are displayed in red whereas those obtained with cells adhered to collagen-coated are displayed in blue. The statistical analysis is here restricted to responding cells. (**A**–**D**)—Average foci intensity over time, the average is computed on responding cells only. (**E**–**H**)—Distribution of foci intensity in responding cells displayed at two time points (1 h and 3 h post HS) by box-and-whisker plot ( whiskers represent 9th percentile and the 91st percentile). (**I**–**L**)—Histogram of the relaxation index, four bins are used: 0≤η<0.25, 0.5≤η<0.75, 0.75≤η<1.25, and 1.25≤η. The relaxation index is defined as the ratio of the foci intensity measured in a given cell at three hours after the stress onset to the one measured one hour after the stress onset.

The number of analyzed cells in each condition (500 cells typically) is sufficient to elaborate a statistical study avoiding the focusing on rare events while preserving the description of the heterogeneity of the population. As we have recently presented, this system performs, for the same culture and hyperthermia condition, a continuum of response [9] that we propose to capture here, for the sake of a simple comparison between culture conditions by four quantities. Firstly, the responsive cell fraction (positive cells), i.e., the cell fraction having a detectable fluorescence foci during the hyperthermia phase (Figure 4). As expected, positive cell numbers increases monotonically with stress intensity, the transition is, however, less abrupt when cultured with the ECM (Figure 4). Secondly, the dynamics of the average intensity, reduced to positive cells only, of the HSF1 fluorescence foci *F* (Figure 5A–D). At each time step the average is calculated only on the positive cells (F>1%). In this way, the amplitude of the response is not affected by the fraction of non-responding cells, which eases comparison of temporal patterns, from one culture condition to another. Thirdly, the distribution, calculated in positive cells and schematized by whisker boxes, of the intensity of the HSF1 fluorescence foci one and three hours after the stress onset (Figure 5E–H). Those distributions quantify the response amplitude heterogeneity, but not the temporal shape heterogeneity (relaxation, plateau, or constant increase). Lastly, the histogram of the relaxation index (Figure 5I–L) describes the temporal shape heterogeneity of the response. A 0% value of this index indicates a perfect adaptation of the signal (*F* increases and then relax towards its original level); a 100% value, a plateau (*F* increases and then maintain its high value); a higher value; a constant increase.

Apart from the differences regarding the number of positive cells already discussed, the responses at 42 °C and 43 °C display strong similarities in both culture conditions either for the average response (Figure 5B,C), the response amplitude distributions (Figure 5F,G) or response shape distributions (Figure 5J,K). The situation is slightly different for the moderate 41 °C and acute 44 °C stresses. If for the former, the mean dynamic suggests a slower relaxation in the absence of the ECM (Figure 5A), it is because the population there exhibits more cells with plateau-like response pattern in the presence of the ECM and no imperfect adaptation, a pattern more present with ECM (Figure 5I). Response heterogeneity at the maximum amplitude, 1h after stress onset, is also greater in the presence of ECM (Figure 5E). The small number of responding cells makes the statistical description at 41 °C more speculative. This is not the case at 44 °C where the majority of cells responds in both culture conditions. In this case, the most significant difference comes from the long time behavior, i.e., 2–3 h after the stress onset. Cells cultured without the ECM indeed show a new increase in the mean fluorescence intensity of foci, where cells cultured with ECM show a plateau (Figure 5H). Again, the average dynamic is not representative of individual dynamics since it masks a combination of plateau and constant increase (Figure 5L). In contrast to the low stresses, the response heterogeneity is here greater without the ECM (Figure 5H).

### 2.4. Mathematical Model Correlates Phenotypic Heterogeneity with Protein Expression Variability

Guilbert et al. [9] present a mathematical model of the response network able to predict the inter dependence between response heterogeneity and expression heterogeneities of the molecular chaperone and the transcription factor shown in Figure 3. Time-lapse microscopy experiments track four cell population samples, one per exposure temperatures, in the same culture condition. Each of these samples supposedly represents the same expression distribution of the molecular chaperone and transcription factor HSF1. Moreover, the total fluorescence distributions of HSF1:eGFP, which are similar across the four samples, confirm that the four samples are indeed representative of the same population.

It is therefore interesting to use this mathematical model to infer the distribution of molecular chaperone expression. In practice, this requires a prior selection of the model parameters that are assumed to vary from cell-to-cell. To focus the attention on the expression levels, we consider that other parameter values are unchanged by the presence or the absence of the ECM, and that they correspond to the previously determined values (reproduced in Table 1).

Therefore, to fit a time trace two free parameters remain, the expression level of molecular chaperones and of the transcription factor HSF1, and to go further, the latter is considered as completely determined by the total fluorescence level of HSF1:eGFP. Thus, the expression level of the molecular chaperone at the initial time remains the only degree of freedom to adjust the response. This approach is quite reductive, because (i) some parameters might depend on the culture condition such as the rate of synthesis; and (ii) HSF1:eGFP fluorescence only imperfectly reflects the total HSF1 expression level, even though HSF1 and HSF1:eGFP expressions are found to be well correlated in the immunofluorescence data.

**Figure 6:**
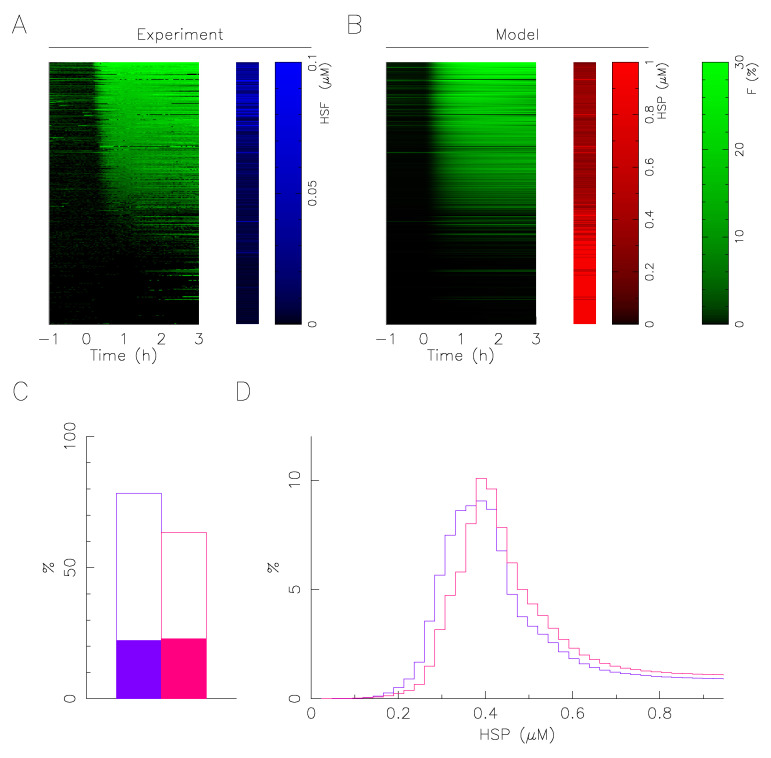
Mathematical modeling predicts HSP expression heterogeneity. (**A**) Fluorescence dynamics of single cell foci (green) and total fluorescence measurement of HSF1:eGFP (blue). Each line corresponds to a single cell (**B**) cell-by-cell best fitting of the fluorescence foci (green) and predicted value of molecular chaperone expression (red). The data correspond to a temperature of 43 °C applied on a population of cells grown on collagen coating. (**C**) Percentage of successful single cell fitting at 43 °C (col+/col−; red/blue). Solid rectangles represent control: no fitting of molecular chaperone expression level, but taking into account HSF1 heterogeneity measured by HSF1:eGFP fluorescence. (**D**) Molecular chaperone expression distribution prediction from the fitting results at 43 °C (col+/col−; red/blue).

Based on this assumption, the mathematical model associates at a single cell the value of expression level of molecular chaperone that fit at best its time series response knowing its total HSF1:eGFP fluorescence (Figure 6). A fit is here considered satisfactory when the average error is less than the error of measurement of the fraction of fluorescence in the foci (2%). Figure 6C represents the percentage of response correctly fitted by considering either the measured heterogeneity of HSF1 only (solid rectangles) or by adding the heterogeneity of the molecular chaperone (open rectangles). This shows how accounting for the heterogeneity of the molecular chaperone helps the response pattern heterogeneity description. However, the HSP determination is only reliable when the response appears, at least transiently, because when the cell does not have foci, a whole range of values at a high level is possible (Figure 6B). Therefore, rather than defining an expression level value (the one corresponding to the best fit), we define a likelihood distribution of the value from the fit score. The sum of the likelihood distributions for a sample thus defines an estimate of the expression distribution of the molecular chaperone in the sample (Figure 6D).

In spite of restrictive working hypotheses (only a single free parameter for the fit) and a large variability of response to describe, the quantitative description of the continuum of responses is very satisfactory, whatever the condition (Figure 6C). The distributions obtained for the expression of the molecular chaperone are all well centered, with a fast growth and a slow decay, similar to a log-normal distribution. The distributions obtained for 43 °C indicate that a slight overexpression of molecular chaperones in the presence of collagen is sufficient to induce the observed smoothing and lesser activation of the response (Figure 3). The obtained overexpression of molecular chaperones in the presence of collagen is in good qualitative agreement with the immunoflurescence measurements. Unfortunately immunoflurescence measure separately the two molecular chaperones (HSP72 and HSC70), and as their relative abundance is not precisely known. It is not possible to deduce quantitatively the evolution of the total population of molecular chaperones.

## 3. Discussion

In a recent study, we showed how a chain of procedures combining quantitative biochemistry, single-cell time tracking and mathematical modeling could be used to highlight, interpret and predict phenotypic heterogeneity of cellular response to proteotoxic stress [9]. Mathematical modeling, based on the well-known regulatory network, specifically allows us to rationalize the large set of experimental data produced by giving experimentally testable clues on the molecular origin of the observed variability. With a similar chain of procedures, we here probe the influence of the cellular environment on phenotypic heterogeneity. If in this first approach we focused on the influence of collagen I, which is the main component of the ECM, and on the response to proteotoxic stress, which has a well-established mathematical model, we believe that the methodology presented is general. The main objective of this work is therefore to study the impact of collagen I coating on the phenotypic heterogeneity of the stress response observed in a monoclonal cell population subjected to environmental heat stress. Our working hypothesis is that the coating induces a modification of the expression levels of proteins, and in particular of those involved in the corresponding regulatory network, which in turn induces a modification of the heterogeneity of the response. To our knowledge, no study has ever been conducted on this issue, although the complexities and importance of ECM [13] and of phenotypic heterogeneity [14] are well characterized, especially in tumor progression and drug resistance where control of heterogeneity is an expected future [15].

As major results, immunofluorescence analysis effectively revealed how the presence of the collagen I coating alters the expression level of the major molecular chaperones of the HSP70 family (HSP72 and HSC70) involved in proteotoxic stress repair (Figure 2). The presence of the coating stretches the distribution of HSF1 expression levels but does not change its median value, whereas it increases the median value of the expression levels of the molecular chaperones (HSP72 and HSC70). The change in the median expression level of molecular chaperones obtained in the presence of collagen I corroborates the known results in the literature [16]. In agreement with our working hypothesis, the dependence of foci on molecular expression levels is not affected by the coating (Figure 3), suggesting that the collagen I affects the molecular expression of the central player of the HSR network and not the internal reaction rate of the HSR network. Let us use an analogy with enzymatic reactions to explain this in a simple framework, the coating affects the enzyme population more than the catalytic rate. High-throughput temporal microscopy provides a complete picture of phenotypic variability with or without a collagen I coating. This massive data was analyzed in two steps: (1) Does the cell respond to stress or not? (2) If it does, how heterogeneous is the temporal response (Figure 5)? Statistical analysis reveals that the presence of the coating has more impact on the fraction of response in a cell population than on the heterogeneity of the temporal response. These puzzling results are fully reconciled by data-driven mathematical modeling. The agreement between the predicted distribution of molecular chaperones (Figure 6) and that observed (Figure 3) suggests the validity of our working hypothesis. Coating alters molecular expression levels, which is sufficient to explain the slight adjustment in phenotypic heterogeneity. Furthermore, this also shows for the first time how a molecular expression distribution can be quantitatively estimated from single cell dynamics using a mathematical model. As minor results, we also showed that expression heterogeneity of molecular chaperones was not related to cell cycle phase, contrary to a regularly raised hypothesis [17].

Nevertheless, several facts that we would like to emphasize here inherently limit the quality of the quantitative description. The first limitation is inherent to the measurement. Indeed, the necessary insertion of a fluorescent reporter for time-lapse microscopy analysis adds a source of noise, only the added protein is indeed quantified and not the wild type one. On the other hand, this insertion does not modify the phenotypic heterogeneity [9]. The second limitation is inherent to the chosen stress network, the multiplicity of molecular chaperones of the HSP70 family, their inducibility or not, their localization in specific compartments, complicates the quantification of expression heterogeneity and thus the connection with the mathematical model which only includes a generic term for this whole family of molecular chaperones. The last limitation comes from the method of inferring, by mathematical modeling, the expression level of the molecular chaperones from the response kinetics. This method, whose principle is exposed here for the first time, could still be mathematically consolidated by determining the estimation error of the expression level distribution from the adjustment errors on the single cell responses.

To go further, it would be very tempting to push the analysis to the cell death to determine if the response heterogeneity observed during the stress phase really explains and predicts the decision taken at much longer times (a few tens of hours here) or if these two processes are decorrelated [18]. As a perspective, the method of inferring molecular chaperone expressions from response kinetics could be experimentally consolidated by measuring this expression level experimentally, either by immunofluorescence performed in situ at the end of the time-lapse microscopy follow-up, or by using an additional fluorescent reporter.

In summary, we show here that the phenotypic heterogeneity of response to proteotoxic stress is not significantly altered by the presence of ECM and that these changes are due to the modification of the expression levels of key proteins of the regulatory network. This result obviously calls for confirmation on other environmental stressors, the methods presented here, combining single cell time lapse microscopy and mathematical modeling will hopefully be inspiring, especially in the field of intra-tumour heterogeneity [14].

## 4. Materials and Methods

Experimental procedure, i.e., cell culture and cell transfection; immunofluorescence staining of HSPs and HSF1; and live/fixed cells imaging; and image processing and analysis are similar to those described in details in Guilbert et al. [9].

### 4.1. Cell Culture and Cell Transfection

The HeLa human cervical cancer cell line (CCL-2^TM^) was purchased from the American Type Culture Collection (ATCC, Manassas, VA, USA). These adherent cells are grown as monolayer in Dulbecco’s modified Eagle’s medium (DMEM; Lonza, Levallois-Perret, France) supplemented with 10% (*v*/*v*) fetal bovine serum (FBS; Life Technologies, Saint-Aubin, France), 1% L-glutamine (2 mM) and 1% (*v*/*v*) penicillin-streptomycin (100 IU/mL) (Lonza). Cell cultures are maintained at 37 °C in a humidified atmosphere containing 5% CO_2_ (*v*/*v*), and passage at preconfluence (twice a week) using 0.05% trypsin-0.53 mM ethylenediamine tetraacetate (EDTA; Lonza). HeLa growing cells are routinely screened for the presence of mycoplasma using DNA-staining with the nuclear dye Hoechst 33342 (1:10,000 dilution) (Sigma-Aldrich, L’Isle d’Abeau Chesnes, France) to avoid collecting data from unknowingly contaminated cell cultures.

Wild-type HeLa cells (HeLa-WT) were transfected with a plasmid expressing the human full-length HSF1 fused to eGFP. The plasmid was kindly provided by Claire Vourc’h (Université Joseph-Fourier, Grenoble, France) and built as previously described [19]. Briefly, PCR amplification allowed to obtain the coding sequence for human HSF1 that was cloned into peGFP N3 vector (Clontech Laboratories, Mountain View, CA, USA); the plasmid was then verified by sequencing (GATC Biotech, Constance, Germany). The transfection (of wild-type HeLa cells with the HSF1:eGFP plasmid) was carried out using FuGENE^®^ HD transfection reagent (Promega, Charbonnières, France) according to the manufacturer’s instructions. The stable HSF1:eGFP-transfected (HeLa-HSF1:eGFP) cell line was then established under selective pressure by 1000 µg/mL geneticin (Life Technologies) followed by selection of a single GFP-positive cell by flow cell sorting system (FACSAria III, Becton Dickinson, San Jose, CA, USA).

All experiments were performed on 2-day-old cell cultures (50% confluence) prepared by seeding 1.8 × 10^5^ cells into 35-mm dishes (Sarstedt, Marnay, France) in complete DMEM without phenol red.

### 4.2. Collagen Coatings

Adherent HeLa cells were seeded on collagen type I coatings into 35-mm dishes (Sarstedt, Marnay, France). To prepare collagen coatings, collagen type I solution from rat tail tendons (Corning; Sigma-Aldrich, L’Isle d’Abeau Chesnes, France) solubilized in 0.018 M acetic acid, was deposited on petri dishes surface at a final concentration of 5 μg/cm^2^. Then, coated substrates were dried overnight under sterile conditions at room temperature, and rinsed with distilled water before cell plating. Wild-type or HSF1:eGFP-stably transfected HeLa cells were then seeded (1.8 ×$10^{5}$ cells/dish) in complete DMEM without red phenol and were grown for 48 h before experiments.

### 4.3. Immunofluorescence Staining of HSPs and HSF1

After 48 h of culture, HeLa-WT and HeLa-HSF1:eGFP cells are heated at 43 °C for one hour in our homemade incubator controlled in temperature and gas conditions [20]. At the end of the thermal stress, and in parallel to the unstressed samples (control, 37 °C), cells are immediately rinsed with Dulbecco’s Phosphate-Buffered Saline (DPBS; Lonza), and fixed in 4% paraformaldehyde in DPBS for 10–15 min at Room Temperature (RT). After washing three-times with DPBS, samples are incubated for 30 minutes at RT in DPBS containing 0.3% Triton X100 and 5% goat serum (*v*/*v*) allowing permeabilization of cells and blocking of non-specific binding sites. Cells are then incubated overnight at 4 °C with monoclonal primary antibody as following: mouse anti-HSC70 (1:100 dilution; Santa Cruz Biotechnology, Dallas, TX, USA), or mouse anti-HSP72 (1:100 dilution; Enzo Life Sciences, Villeurbanne, France), or rabbit anti-HSF1 antibody (Enzo Life Sciences). Subsequently, samples are washed, and incubated for 90 min at RT with either a goat anti-mouse (for HSC70/HSP72 expression) or a goat anti-rabbit (for HSF1 expression) secondary antibody conjugated to Alexa Fluor-594 (Life Technologies). A DNA-staining using Hoechst 33342 (1:10,000) is also performed for all samples, to allow the automatic detection of nuclear areas for image analysis.

### 4.4. Live/Fixed Cells Imaging

Either for fluorescence or brightfield images, Hela cells are placed on an inverted microscope (Nikon TiE) with a 40× objective (NA = 0.6). In order to have statistically reliable results, samples are placed on a XY-motorized stage in order to image and track around 500 cells for a given condition. For time-lapse experiments, cells are placed in a temperature and atmosphere regulated incubator and imaged every ten minutes to follow nuclear stress bodies kinetics at the single cell level.

### 4.5. Image Processing and Analysis

All image processing and data analysis was performed using custom written algorithms either in Fortran. Cells were automatically segmented using brightfield image z-stack. Image segmentation was visually inspected after image processing. Corrections to cell segmentation were carried when necessary via a custom written semi-automated graphical interface by either removing false positive or correcting masks. After cell segmentation cells were tracked by linking the closest cell found in the next image. Visual inspection of the tracking did not reveal errors as the cells do not move significantly in the time interval between two acquisitions. We then estimate background for fluorescence images by convolving the raw data with a 30 pixels wide gaussian kernel (larger than cell size) and averaging across the z-stack. Background was subtracted to raw data for further analysis. Total HSF1:eGFP intensity was simply estimated by integrating fluorescence intensity over the whole cell mask. HSF1:eGFP foci were automatically detected by use of standard spatial filters for blob detection (Laplacian of Gaussian). The *F* factor was defined as the integrated intensity found in all foci divided by the total cellular fluorescence. The detection limit for foci is set at 1% of HSF1:eGFP fluorescence localized in foci.

For fixed cells, immunofluorescence experiments image segmentation was achieved on images from HSP fluorescence channel for whole cell segmentation and on the images from Hoechst fluorescence channel for the nucleus segmentation. We acquired fluorescence images of dishes filled with fluorescent dye for flat field correction. The dyes were courmarin for Hoechst channel, rhodamine 110 for GFP and AlexaFluor488 channel and rhodamine B for AlexaFluor594 channel. After flat field correction images were segmented using a modified Otsu thresholding method. A constant background was subtracted before further analysis. *F* was defined as above and HSP concentration was defined as the total fluorescence inside nucleus divided by the nuclear area in arbitrary units.

### 4.6. Mathematical Model for HSRN

The mathematical model describing the cellular response to heat stress has been previously described and calibrated [9], its equations read

(1a) $$\begin{array}{cc} \tau_{\theta }\;\frac{d}{dt}\theta = & \theta_{c}-\theta \end{array}$$

(1b) $$\begin{array}{cc} \tau_{\mathrm{MFP}}\;\frac{d}{dt}\left[\mathrm{MFP}\right]= & \kappa \left(\theta \right)-\frac{{\left[\mathrm{MFP}\right]}^{2}}{\left[\mathrm{HSP}]+[\mathrm{MFP}\right]}-k_{r}\frac{\left[\mathrm{MFP}]\;[\mathrm{HSP}\right]}{\left[\mathrm{HSP}]+[\mathrm{MFP}\right]} \end{array}$$

(1c) $$\begin{array}{cc} \tau_{\mathrm{HSP}}\;\frac{d}{dt}\left[\mathrm{HSP}\right]= & \beta \;\frac{{\left[\mathrm{HSP}\right]}_{\mathrm{free}}}{H_{0}+{\left[\mathrm{HSP}\right]}_{\mathrm{free}}}\;\left[\mathrm{mHSP}\right]-\left[\mathrm{HSP}\right] \end{array}$$

(1d) $$\begin{array}{cc} \tau_{\mathrm{mHSP}}\;\frac{d}{dt}\left[\mathrm{mHSP}\right]= & \mu +\lambda \frac{{\left({\left[\mathrm{HSF}1\right]}_{\mathrm{free}}\right)}^{3}}{S_{0}^{3}+{\left({\left[\mathrm{HSF}1\right]}_{\mathrm{free}}\right)}^{3}}-\left[\mathrm{mHSP}\right] \end{array}$$

(1e) $$\begin{array}{cc} {\left[\mathrm{HSP}\right]}_{\mathrm{free}}= & \frac{\left[\mathrm{HSP}\right]}{\left[\mathrm{HSP}]+[\mathrm{MFP}\right]} \end{array}$$

(1f) $$\begin{array}{cc} {\left[\mathrm{HSF}1\right]}_{\mathrm{free}}= & \frac{{\left[\mathrm{HSF}1\right]}_{\mathrm{tot}}}{{\left[\mathrm{HSF}1\right]}_{\mathrm{tot}}+\left[\mathrm{HSP}\right]\;\frac{\left[\mathrm{HSP}\right]}{\left[\mathrm{HSP}]+[\mathrm{MFP}\right]}} \end{array}$$

where *t* is time; θ is the temperature of the cell environment measured in ° [MFP], the misfolded protein concentration; [mHSP], the concentration of mRNA coding for HSP; [HSP], the heat shock protein concentration; ${\left[\mathrm{HSF}1\right]}_{\mathrm{tot}}$ the heat shock factor 1 protein total concentration. The denaturation rate κ(θ) is here the only temperature input [11]:

(2) $$\kappa_{d}\left(\theta \right)=k_{d}\;\left(1-0.4e^{37-\theta }\right)\;1.4^{\theta -37}$$

The signification and values of model parameters are summarized in Table 1. The fluorescence fraction within foci is described by:

$$F={\left({\left[\mathrm{HSF}1\right]}_{\mathrm{free}}\;/\;{\left[\mathrm{HSF}1\right]}_{\mathrm{tot}}\right)}^{3}$$

For the parametric estimation of the HSP concentration in each cell, the total HSF1:eGFP fluorescence of each cell is used to determine the parameter ${\left[\mathrm{HSF}1\right]}_{textrmtot}$. For this, a proportionality relation is assumed between the fluorescence of HSF1:eGFP and the concentration of HSF1 in each cell, the conversion factor is obtained by making the average value of the fluorescence intensity cohere to the reference value of the model established in [9] and reported in the Table 1. This fluorescence renormalization procedure is also used for the representation in Figure 6A. The value of the initial concentration of HSP, [HSP](t=0), is then freely variable. The duration of the stress being much shorter than the characteristic time of evolution of HSP, we have chosen for simplicity to vary the initial concentration rather than the value of the model parameters. This facilitates the direct comparison with the biochemical measurements presented in Figure 3.

## Figures and Tables

**Table 1:** Estimated Parameter of the heat shock response network.

Parameter	Unit	Description	Value
$k_{d}$	(μM)	denaturation rate	1.76
$k_{r}$		renaturation rate	17.7
μ	(μM)	HSP basal transcription rate	1.47 × $10^{-3}$
λ	(μM)	HSP active transcription rate	0.78
$S_{0}$	(μM)	HSP transcription regulation threshold	0.18
β		HSP translation rate	10
$H_{0}$	(μM)	translation regulation threshold	0.32
${\left[\mathrm{HSF}1\right]}_{\mathrm{tot}}$	(μM)	HSF1 concentration	4.0 × $10^{-2}$
$\tau_{Temp}$	(h)	incubator rise time	1/15
$\tau_{MFP}$	(h)	MFP lifetime	0.5
$\tau_{mHSP}$	(h)	mHSP lifetime	1
$\tau_{HSP}$	(h)	HSP lifetime time	10

## Data Availability

The data presented in this study are available on request from the corresponding author.

## Abbreviations

The following abbreviations are used in this manuscript:

ECM	ExtraCellular Matrix
HSP70	70-kDa Heat Shock Protein family
HSP72	Heat shock 70 kDa protein 1 (HSPA1A)
HSC70	Heat shock 70 kDa protein 8 (HSPA8)
HSF1	Heat shock factor 1
NT	NormoThermia
HT	HyperThermia
F	Fraction of HSF1 fluorescence within foci
